# Field Evaluation of the Bio-Efficacy of Three Pyrethroid Based Coils against Wild Populations of Anthropophilic Mosquitoes in Northern Tanzania

**DOI:** 10.4103/0974-777X.62885

**Published:** 2010

**Authors:** Shandala Msangi, Beda J Mwang'onde, Aneth M Mahande, Eliningaya J Kweka

**Affiliations:** *Tropical Pesticides Research Institute, Division of Livestock and Human Disease Vector Control, P. O. Box 3024, Arusha – Tanzania*

**Keywords:** Bio-efficacy, Kiboko^®^, Mosquito-coils, Risasi^®^, Total^®^, Tanzania

## Abstract

**Aims:**

This study aims to assess the feeding inhibition and repellency effect of three brands of mosquito coils in experimental huts (East African design). Evaluated products were all pyrethroid-based mosquito coils–Kiboko®, Total® and Risasi®. Mosfly (0.1% D-allethrin) was a positive control. Indoor resting behavior, feeding inhibition and induced exophily were measured as responses of burnt coil smoke in huts.

**Materials and methods:**

Resting mosquitoes were collected inside the huts, in window traps and verandah traps using mechanical aspirators. Identified to species level and sex.

**Results:**

A total of 1460 mosquitoes were collected, 58.9% (n=860) were *Anopheles gambiae s.l* while 41.1% (n=600) *Culex quinquefasciatus*. Indoor resting mosquitoes in all treated huts were significantly reduced than in negative control (DF=4, F=18.6, *P* < 0.001). Species found to rest indoors were not statistical different between the positive control (Mosfly coil) and other three treated huts (DF=3, F=1.068, *P=*0.408). *Cx.quinquefasciatus* had significantly higher induced exophily in all treatments comparing to *An.gambiae s./* (DF=1, F=5.34, *P=*0.050). Comparison between species (*An.gambiae s.l* and *Cx. quinquefasciatus*) for the feeding inhibition among treated huts was not statistically significant (DF=1, F=0.062, *P*=0.810).

**Conclusion:**

Introduction of several personal protection measures will be ideal to supplement the existing gap in reducing the man vector contacts hence lowering the disease transmission.

## BACKGROUND

Vector-borne diseases are responsible for 17% of the estimated global burden of infectious disease.[1] *Plasmodium falciparum*, a mosquito transmitted malaria parasite, is responsible for 350 to 500 million clinical malaria cases and over 1.7 million deaths annually.[2,3] In Tanzania, twenty-eight million citizens are exposed to risk of stable malaria causing 16 million clinical cases and one hundred thousands child deaths with over 25% of total death.[4,5] In a state of emergence where there is no well planned program for vector control measures such as insecticides treated bed nets (ITNs) and long lasting insecticide nets (LLINs);[6,7] use of both synthetic and plant based repellents[8–10] and use of indoor residual spray,[11] mosquito coils can be deployed. Also, the house design improvements have been considered as the physical barrier tool for indoor mosquito reduction.[12] The use of mosquito coils has been gaining popularity in communities with both high and low malaria transmission intensities as a supplement for protection(meanwhile indoor but outside bed net).[13,14] But the coverage of the ITNs in community has been highly affected by social economic status and availability in rural areas.[13,15] The bio-efficacy of mosquito coils impregnated with synthetic pyrethroids against mosquito have been reported in other studies.[16,17] However, efficacy of different and new coil formulations may need to be evaluated in different settings. It was therefore the objective of this study to evaluate three brands of pyrethroid based mosquito coils in the field for its feasibility in community use against mosquito house entry and biting.

## MATERIALS AND METHODS

### Study area

The work was conducted between February to March 2008 using Experimental Hut at Magugu Field Station of the Tropical Pesticides Research Institute in Northern Tanzania (4' 00 S, 35' 46 E). The use of experimental huts and the biological tests involved in testing efficacy of various insecticides against anthropophilic mosquitoes is well described by Smith,[18] Smith and Webley[19]

### Evaluated products

The tested products were all pyrethroid based mosquito coils–Kiboko® with 0.15% D-allethrin imported by MSK Industries Ltd of Dar es Salaam, Tanzania. The second product was Total® with 0.2% D-allethrin, imported by Total Tanzania Ltd. The third product was Risasi®, a mosquito coil which is a botanical based insecticide/repellent, with 0.2% w/v pyrethrins as its active ingredient imported and distributed by Meghji Sundries of Dar-Es-Salaam, Tanzania. Mosfly (0.1% D-allethrin) was used as a standard for comparison purpose because it was a registered mosquito coils in Tanzania.

### Experiment design

Five experimental huts (East African design) were used in this study. Three huts with mosquito coil treatments and two served as negative and positive control in a 5×5 Latin square design. The three mosquito coils were randomly allocated to the huts. Two huts remained as negative and positive controls. In the experimental huts, mosquito coils were supported by their stands, placed distantly from a bed occupied by a volunteer with untreated bed net. The coils were lit at 20.00 hours and left to bum out, burning duration was recorded for each coil type. Mosquitoes from inside the huts, window and verandah traps were collected and recorded as described in other studies.[19,20] Experiments were repeated by systematic rotation of coils in huts to avoid hut positional and individual attractiveness biasness. The treatments were rotated in two days after first experimental rotation to avoid contamination in the hut due to coils effect.

### Mosquito collection

From 06:30 to 07:30 hours in the morning, mosquitoes were collected from indoors, verandah and window traps of the huts. Collections were done by a pair of trained mosquito collectors using hand aspirators.[20] Mosquitoes collected were put separately in paper cups and later sorted into their species and abdominal conditions.[21] Males were ignored during collections.

### Ethical approval

The study was approved by the Tropical Pesticides Research Institute, Research Ethics Committee. The oral and written informed consent was obtained from the sleepers to participate in the study before getting involved in experiments. Malaria screening were done weekly and treatments were given to any person with malaria parasite free of charge, but fortunately none of volunteer had malaria parasites during and two weeks after study.

### Statistical analysis

Mosquitoes collected from experimental and control huts were compared using SPSS program version 15.0 for windows. General linear model univariate was performed to assess the effect of the factors in experiments. Days, volunteers, huts and coils were analyzed to assess the effect of each in experimental design. Significance level was considered at Probability (*P*) <0.05.

### Indoor resting mosquitoes

The mean numbers of mosquitoes collected in the treated huts were compared with the mosquitoes from positive and negative control huts. Mosquitoes collected were compared by day, hut, treatments and volunteers.

### Induced exophily (deterrence/repellency effect of coils)

The mosquitoes found in verandah and window traps were considered escaping the excito-repellency/irritant effect of the coils smoke in the hut and therefore were used to estimate repellency/deterrence effect of the coils. The mean numbers of unfed mosquitoes from verandah and window traps were compared separately with the untreated hut mosquitoes from verandah and window traps respectively. The induced exophily was calculated using Abbott formula {(N_c_ – N_t_)/N_c_} × 100% where N_t_ is a number of mosquitoes from verandah and window traps of treated hut while N_c_ is a number of mosquitoes from verandah and window traps of untreated hut.

### Mosquitoes feeding inhibition

The mean number of fed mosquitoes collected indoors, verandah trap and window trap were subjected to ANOVA for statistical differences between treated and untreated huts. The percentage inhibition was also calculated using Abbott formula,[22] {(F_c_ – F_t_)/F_c_} × 100% where F_c_ is a number of mosquitoes found fed in untreated hut while F_t_ is a number of mosquitoes fed in treated hut.

## RESULTS

### Catches of *An. gambiae s.l* and *Cx. quinquefasciatus*

During the 25 days of experiments, a total of 1460 mosquitoes were collected in indoor, verandah and window traps of the five huts. Among those 58.9% (Number of mosquitoes (N)=860) were *An.gambiae s.l* while 41.1% (N=600) were *Cx. quinquefasciatus.*

### Indoor resting mosquitoes

**Figure 1:**
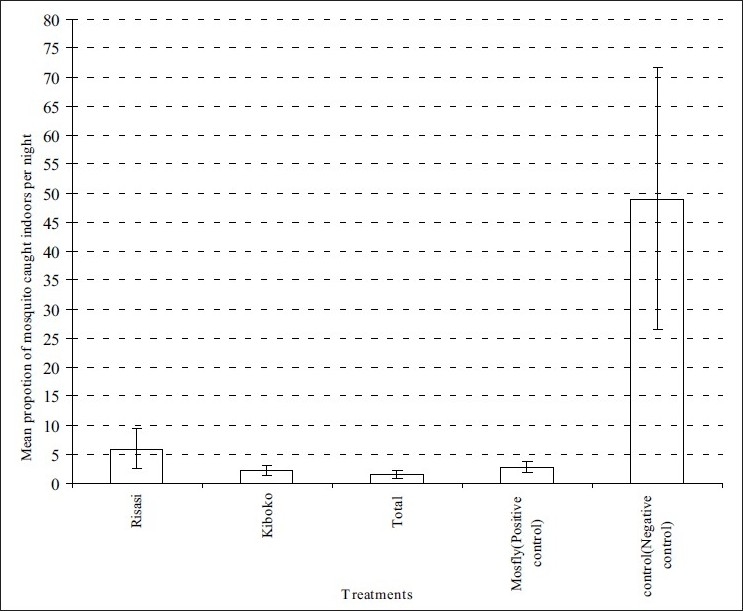
Mean proportion of mosquitoes resting indoors in each treatment

In analysis, other factors (day, hut, and volunteers) had no effect on experiment except mosquitoes populations caught indoor in each treatment. The total populations of indoor resting mosquitoes in all treated huts were significantly reduced in comparison to negative control (Degree of Freedom {DF}=4, F-Test result (F)=18.6, Probability (*P*)<0.001) as shown in Figure 1. The species found to rest indoors were not statistically different between the positive control (Mosfly coil) and other three treated huts (DF=4, F=1.068, P=0.408). (F- Indicate result of F-test which was used to compare difference between means of the collected mosquito samples)

### Induced exophily (deterrence/repellency effect of coils)

The induced exophily for the *Cx.quinquefasciatus* was 92%, 96%, 96%, 96% and 0% while for the *An.gambiae s.l* was 63%, 64%, 60%, 60% and 0% for Mosfly, Risasi, Total, Kiboko and negative control respectively. The statistical comparison of the two species showed that, *Cx. quinquefasciatus* had significantly higher induced exophily comparing to *An.gambiae s.l* in all treatments (DF=1, F=5.34, P=0.050).

### Feeding inhibition

In all treatments, the feeding inhibition for the *Cx.quinquefasciatus* was 100%, 96%, 91%, 100% and 0% while for the *An.gambiae s.l* was 100%, 59%, 100%, 94% and 0% for Mosfly, Risasi, Total, Kiboko and negative control respectively. Comparison between species (*An.gambae s.l* and *Cx.quinquefaciatus*) for the feeding inhibition among treated huts was not statistically significant (DF=1, F=0.062, P=0.810).

### Mortality

In all experimental huts, among *Cx.quinquefasciatus* and *An.gambiae* *s.l* collected, none died after being held for twenty-four hours of observation in provision of 10% sugar solution.

## DISCUSSION

This study has proven the effectiveness of mosquito coils as personal protection tool for malaria vectors and nuisance mosquitoes based on mosquito behavior, which is in accordance with previous studies.[16] In this study, the parameters which were aimed to measure the impact of coils in reducing human vector contact such as feeding inhibition, induced repellency (deterrence) and indoor resting behavior were evaluated. In assessing feeding inhibition, all coils did better against the two species except Risasi which protected only 59% of *An.gambiae s.l* from feeding in eight hours time. In induced exophily for *An.gambiae s.l,* Total and Kiboko gave a repellency of 60% while Risasi gave 63% in *An. gambiae s.l* while in *Cx. quinquefasciatus* all evaluated coils induced exophily above 90% relative to the negative control. These observations suggest that low levels of pyrethrins cause repellency to mosquitoes, including feeding inhibition and induced exophily as concluded by other workers[16,23] hence reduction in disease transmission.[14] As found by MacIver,[23] the low level of pyrethrin have been considered to have no knockdown effect unless the concentration of pyrethrin increased. The current investigations of mosquito coils have yielded similar observations that, pyrethrins at lower dosages were more efficient in repellency as observed in other studies.[24,25] The factors evaluated suggest the possibilities of recommending these coils to be used in urban and rural areas where culicines and anophelines predominate respectively. The evaluated coils were found to have more than 75% feeding inhibition for *An.gambiae s.l,* the malaria vectors which are similar to results recommended for personal protection tool to be effective in reducing disease transmission in community level.[6]

From these results, we therefore recommend the use of pyrethroid based mosquito coils in complementing the existing mosquito control measures such as Indoor Residual Sprays (IRS) and Insecticide Treated Nets (ITNs), especially in rural area where the disease is endemic.[11,12,14] Malaria vectors and nuisance biting mosquitoes in most of African disease endemic areas have developed resistance against commonly used insecticides for treating bed nets.[26–28] In Tanzania insecticides resistance genes towards pyrethroids; most used insecticides is still at very low frequencies.[29] the area were these mosquitoes coils were evaluated malaria vectors are susceptible to pyrethroid insecticides used.[29,30] Resistance to available insecticides has posed a major threat in vector control and more efforts have to be done in implementation of integrated vector control management practices.

The burning duration for these coils was eight hours which was reported to be similar to other studies done in Tanzania with pyrethroid coils.[16] The burning duration covers the active mosquito biting cycles and therefore acceptable in malaria control programs in supplementing the existing control tools for community use. Moreover, the low costs of most mosquito coils, as compared to other tools, make this mosquito control tool appropriate for low income rural communities and in areas with emergence control needs such as refugee camps. There were no complaints from the volunteers in experimental huts in respect to the side effects of the coils as reported from other mosquito-coil studies.[31,32] The proper use of mosquito coils will complement the existing tools for personal protection against infective bites of malaria and nuisance mosquitoes.

Despite the great achievements of this study, the main constraints during the experiments were during mosquito-collection, which sometimes was done when other people were still on bed. The study duration was restricted by the number of mosquito coils provided by factories for evaluation.

## CONCLUSION

It can be concluded from the findings of this study that, pyrethroid-based mosquito coils when properly used and coupled with other tools such as indoor residual spray and insecticides treated bed nets can have an impact in reducing indoors vector-human contact.
